# The role of LLPS of biomacromolecules in the gonads and related diseases

**DOI:** 10.3389/fendo.2026.1800034

**Published:** 2026-09-28

**Authors:** Hui Zhao, Xiaodong Wu, Yuqing Chen, Dandan Luo, Qingbo Guan, Chunxiao Yu, Peng Lu

**Affiliations:** 1 Key Laboratory of Endocrine Glucose & Lipids Metabolism and Brain Aging, Ministry of Education, Department of Endocrinology, Shandong Provincial Hospital Affiliated to Shandong First Medical University, Jinan, Shandong, China; 2 Department of Endocrinology, Shandong Provincial Hospital, Shandong University of Traditional Chinese Medicine, Jinan, Shandong, China; 3 Weihai Municipal Hospital, Shandong University, Weihai, Shandong, China; 4 Shandong Key Laboratory of Endocrinology and Lipid Metabolism, Shandong Institute of Endocrinology and Metabolic Diseases, Shandong Engineering Laboratory of Prevention and Control for Endocrine and Metabolic Diseases, Jinan, Shandong, China; 5 “Chuangxin China” Innovation Base of Stem Cell and Gene Therapy for Endocrine Metabolic Diseases, Shandong Engineering Research Center of Stem Cell and Gene Therapy for Endocrine and Metabolic Diseases, Jinan, Shandong, China

**Keywords:** biomolecular condensates, gonads, LLPS, membraneless organelles, reproductive system, reproductive tumors

## Abstract

Liquid–liquid phase separation (LLPS), an emerging mechanism of intracellular organization and regulation, has attracted increasing attention in reproductive biology in recent years. By promoting the spatiotemporal compartmentalization of biomolecules, LLPS is involved in a wide range of essential biological processes, including transcriptional regulation, chromatin remodeling, and mRNA stabilization and translation. Increasing evidence has demonstrated that LLPS plays indispensable roles in the development, maturation, and differentiation of both sperm and oocytes, while also contributing to the initiation and progression of sex hormone-dependent tumors. Despite these advances, the mechanistic links and overarching roles of LLPS in both gametogenesis and sex hormone-dependent tumorigenesis have not yet been comprehensively summarized. A clearer understanding of the functions and regulatory mechanisms of LLPS in gamete development and tumorigenesis will not only deepen our insight into the complex molecular networks underlying germ cell biology, but also offer potential molecular targets and a conceptual basis for the diagnosis and treatment of reproductive system disorders.

## 1 Basic principles and characteristics of LLPS

In the 1830s, scientists identified a distinct structure within the nuclei of nerve cells that was later termed the nucleolus, one of the earliest recognized membraneless compartments (1). Subsequent studies revealed many structurally and functionally related compartments throughout eukaryotic cells, including in the nucleus and cytoplasm, which are now collectively referred to as phase-separated membraneless organelles (PMLOs) (2). Unlike classic membrane-bound organelles such as the endoplasmic reticulum and Golgi apparatus, PMLOs lack a surrounding lipid bilayer and therefore cannot compartmentalize molecules or regulate their exchange through conventional membrane transport mechanisms (3). A major breakthrough came in 2009, when Brangwynne and colleagues demonstrated that P granules in the germ cells of Caenorhabditis elegans embryos are dynamic liquid-like structures showing fusion, dissolution, deformation, and rapid exchange with the surrounding environment. This finding provided the first strong evidence that certain membraneless intracellular compartments can assemble through LLPS, thereby opening a new avenue of research in this field (4). Since then, LLPS has been recognized as a central physical principle underlying the formation of many membraneless cellular compartments. By concentrating proteins and nucleic acids into highly hydrated and dynamic microenvironments, LLPS can enhance local reaction efficiency and has also been discussed in the context of primitive compartmentalization and early life-like systems (5).

The LLPS of proteins is primarily driven by multivalent interactions mediated by intrinsically disordered regions (IDRs) and/or modular domains—that is, the ability to engage in multiple weak and reversible interactions that support rapid assembly, disassembly, and reorganization. IDRs frequently contain low-complexity domains (LCDs), whose amino acid composition is often enriched in specific types of residues, thereby enhancing their multivalent interaction capabilities (6). However, LLPS is not restricted to IDR-driven interactions alone; it can also arise from multivalent interactions among ordered domains or between proteins and nucleic acids, such as the binding between SRC-homologous 3 (SH3) domains and proline-rich motifs (7). One of the defining characteristics of LLPS is that its formation depends on a specific concentration threshold, known as the saturation concentration. Unlike the progressive formation of stoichiometric protein complexes as the concentration of a single component increases, LLPS occurs only when the component concentration exceeds this threshold. This threshold is regulated by multiple environmental factors, including temperature, ionic environment, pH, the presence of cosolutes, and excluded-volume effects exerted by other macromolecules (8–10).

With advances in modern cell biology and microscopic imaging techniques, researchers have discovered numerous membraneless biomolecular aggregates within cells. These structures, typically composed of proteins and RNA, are collectively termed ribonucleoprotein (RNP) particles (2). Representative RNP particles include nuclear bodies such as Cajal bodies, nucleoli, and nuclear speckles, as well as cytoplasmic structures such as P-bodies and stress granules (SGs) (11). In addition to classical RNP granules, phase-separated assemblies also participate in signal transduction, as illustrated by Dishevelled (Dvl) condensates in the Wnt signaling pathway and signaling clusters formed during T cell activation (12). Although highly dynamic, these condensates can maintain characteristic sizes and spatial organization while continuously exchanging components with the surrounding cytoplasm or nucleoplasm. Their distinct molecular composition and localization enable them to regulate diverse biological processes, including DNA replication, RNA processing and transport, signal transduction, synaptic activity, and cellular stress responses (13). Under various stress conditions, such as exposure to heat shock, oxidative stress, ultraviolet irradiation, osmotic stress, and nutrient deprivation, SGs are generated. As mentioned earlier, SGs are membraneless RNP complexes primarily composed of untranslated mRNA, the 40S ribosomal subunit, RNA-binding proteins (RBPs), and factors involved in translation initiation. Aberrant SG assembly has been implicated in a variety of severe diseases (14–16). In addition, work by Adriano Aguzzi and colleagues suggests that LLPS can drive the transition of dynamic liquid condensates into more stable pathological aggregates, thereby disrupting normal cellular function and promoting disease development (17).

In recent years, LLPS has been extensively investigated both *in vitro* and *in vivo*. Common hallmarks of LLPS include spherical morphology, fusion behavior, and fluorescence recovery after photobleaching (FRAP), reflecting dynamic internal molecular exchange (10). *In vitro*, the most traditional approach is the direct microscopic observation of droplet formation and behavior under controlled conditions, such as changes in salt concentration, pH, temperature, or RNA content (18). FRAP is frequently used to assess molecular mobility within condensates (19). Additionally, 1,6-hexanediol treatment can help evaluate the contribution of weak hydrophobic interactions to condensate stability, as 1,6-HD has been shown to promote protein droplet dissolution by stabilizing hydrophobic amino acid residues (20). In living cells, optogenetic systems mediated by photosensitive proteins enable localized phase transitions and provide a useful strategy for investigating LLPS behavior *in vivo* (21). Collectively, these insights and methodologies offer a robust framework for exploring the roles of LLPS in reproductive endocrinology, gametogenesis, and sex hormone-dependent tumorigenesis.

## 2 Physiological roles of LLPS in gametogenesis and gonadal function

### 2.1 LLPS as a conserved organizing principle in reproductive biology

In the human reproductive system, the gonads function as both sites of gametogenesis and essential endocrine organs. They are responsible for producing gametes and for synthesizing and secreting sex hormones required to maintain reproductive capacity and hormonal homeostasis (22, 23). Although male and female gonads are anatomically distinct, they share these dual functions. Importantly, LLPS should not be regarded as a process unique to reproductive cells. Rather, it represents a broadly conserved principle of cellular organization and plays critical roles in diverse cellular activities, including gene transcription, protein translation, chromatin organization, signal transduction, immune responses, autophagic degradation, and cytokinesis (24). This broad relevance is supported by studies in non-reproductive cell systems, including neurons and immune cells. In neurons, LLPS has been implicated in the organization of synaptic compartments, including phase separation-mediated postsynaptic density formation at excitatory synapses, LLPS-dependent clustering of synaptic vesicles, and condensate-mediated active zone assembly in presynaptic boutons (25). In immune cells, LLPS has been linked to the spatial organization and amplification of T cell receptor signaling, as exemplified by LAT and its binding partners undergoing multivalent assembly and phase separation to form liquid-like clusters that promote downstream MAPK/ERK signaling (26).

Thus, reproductive cells, including germ cells, are unlikely to be unique in their reliance on LLPS; rather, gametogenesis provides a physiologically important context in which broadly conserved LLPS-based mechanisms are deployed to coordinate RNA storage, translational timing, chromatin remodeling, and cellular differentiation.

### 2.2 Evidence strength and conceptual boundaries of LLPS in gonadal function

In this context, current evidence most strongly supports a role for LLPS during germ cell development, particularly in RNA storage, translational regulation, germ granule assembly, and chromatin remodeling. In contrast, direct evidence for LLPS-mediated mechanisms in hormone-producing gonadal somatic cells, such as Leydig cells in the testes and granulosa or luteal cells in the ovaries, remains limited. Therefore, the involvement of LLPS in gonadal endocrine regulation should currently be considered an emerging hypothesis rather than an established mechanism.

However, mechanistic studies in hormone-responsive non-gonadal models and hormone-dependent tumor cells provide proof-of-principle evidence that LLPS-associated condensates may serve as regulatory interfaces linking ligand-activated receptors, transcriptional co-regulators, and hormone-responsive genomic elements. For example, glucocorticoid receptor condensates have been linked to DNA-dependent receptor dimerization, co-regulator enrichment, and transcriptional transactivation; GR foci have also been shown to require interactions with specific chromatin regions and to contain Mediator components. Similarly, androgen receptor (AR) condensates recruit coactivators such as MED1 at super-enhancer regions and regulate AR target-gene expression, whereas estrogen-activated enhancers assemble phase-separated enhancer RNA-dependent ribonucleoprotein complexes that promote cooperative chromosomal enhancer activation (27–30). These findings support the concept that LLPS can organize steroid receptor–genome interactions in selected experimental systems, but they do not yet establish that analogous mechanisms operate in gonadal endocrine somatic cells.

More broadly, several fundamental properties of LLPS make it particularly relevant to reproductive biology. These include its dynamic and reversible nature, its ability to concentrate and protect RNAs and proteins in the absence of membrane boundaries, and its sensitivity to developmental and environmental cues. Together, these features are especially well suited for germ cells, which must coordinate transcriptional silencing, RNA storage, stage-specific translation, and large-scale cellular remodeling with high temporal precision (31).

Because current evidence for LLPS in reproductive biology has been obtained from multiple model systems (**Table 1**), including mammals, Caenorhabditis elegans, Drosophila melanogaster, and other vertebrate or invertebrate models, we identify the species or experimental system when discussing individual examples below. This distinction is important because the strength and physiological relevance of the available evidence vary substantially among species and experimental systems. Accordingly, non-mammalian studies are used below primarily to define mechanistic or biophysical principles of biomolecular condensation, whereas conclusions regarding mammalian and human reproductive physiology are based preferentially on evidence obtained directly from mammalian systems. Where corresponding mammalian or human data are unavailable, the cross-species relevance of these mechanisms is considered provisional rather than established. Accordingly, in the following subsections, we summarize the role of LLPS in gametogenesis and discuss its putative impact on gonadal endocrine regulation, while highlighting the need for further studies in the endocrine context.

**Table 1:** Comparative landscape of biomolecular condensation in germ cell development and gonad physiology across species models.

Compartment/condensate	Species	Cellular context	Primary driver(s) & Associated scaffolds	Biophysical state & Material properties	Target physiological processes	Primary key references
Intermitochondrial Cement (IMC)	Mouse	Pachytene-stage male germ cells	TDRD1, MILI, sDMA-modified proteins	Dynamic liquid-like coacervate; high internal mobility	piRNA biogenesis, transposon silencing, mitochondrial clustering	Gao et al., 2024 (44)
Paternal mRNP Condensates	Mouse	Late-stage spermatocytes & postmeiotic spermatids	FXR1, EIF4G3, PABPC1	Liquid-like ribonucleoprotein (RNP) droplets	Stage-specific translational activation of stored paternal mRNAs	Kang et al., 2022 (45)
CEP112 Translational Complex	Human & Mouse	Developing spermatogenic cells	CEP112, EIF4A1, EEF1A1, hnRNPA2B1	Liquid-like RNP condensates	Translational gating of essential spermiogenesis genes	Zhang et al., 2024 (46)
Nuclear CCER1 Condensates	Mouse & Human genetic evidence	Postmeiotic spermatids	CCER1	Nuclear phase-separated foci; highly concentrated	Histone-to-protamine transition, chromatin compaction (TSLN-independent)	Qin et al., 2023 (47)
Balbiani body (Bb)	Zebrafish & *Xenopus*	Early oogenesis	Bucky ball (Buc) [Zebrafish], Xvelo [Frog] (both possessing Prion-Like Domains)	Viscoelastic liquid-to-solid transition; stable amyloid-like matrix (developmentally reversible)	Mitochondrial clustering, maternal RNA storage, animal-vegetal polarization	Kar et al., 2025 (55); Boke et al., 2016 (118)
Mitochondria-Associated Domain (MARDO)	Mouse	Mammalian oocytes	ZAR1	Mitochondria-associated hydrogel-like matrix	Stabilization of maternal mRNAs, large-scale mitochondrial clustering	Cheng et al., 2022 (52)
liquid-like spindle domain (LISD)	Mouse	Mammalian oocytes	TACC3, AURKA, CHC17, microtubule-associated regulators	Highly dynamic, liquid-like microtubule-associated domain	Acentrosomal spindle assembly, chromosome alignment & segregation	So et al., 2019 (56)
Cytoplasmic P-Bodies	*Drosophila*	Mature/Arrested oocytes	Me31B, Tral	Dense, aggregated RNP granules	Storage of bicoid (bcd) mRNA; regulated release after egg activation; early embryonic patterning	Sankaranarayanan et al., 2021 (51)
Paraspeckle Nuclear Bodies	Mouse	Ovarian corpus luteum	Neat1 (lncRNA), NONO	Block-copolymer micellar core-shell structures	Corpus luteum formation/function, progesterone production, establishment of pregnancy	Nakagawa et al., 2014 (59); Yamazaki et al., 2021 (119)

### 2.3 LLPS-associated RNA granules in male germ cell development

During early embryonic development, primordial germ cells (PGCs) are specified from pluripotent embryonic cells and migrate to the genital ridges, thereby establishing the germline lineage. In mammals, PGC-derived prospermatogonia/gonocytes subsequently give rise to spermatogonial stem cells (SSCs), which provide the cellular basis for spermatogenesis. In mice, this transition occurs during early postnatal development, when gonocytes re-enter the mitotic cycle around postnatal days 3–4 to establish the SSC pool. In response to specific signaling cues, SSCs sequentially differentiate into type A, intermediate, and type B spermatogonia. These cells then undergo mitotic division to produce primary spermatocytes, which subsequently complete two rounds of meiosis to generate haploid spermatids. Finally, through extensive morphological remodeling, spermatids differentiate into mature spermatozoa (32).

This sequential germline differentiation and morphological transition, along with the precise stage-aware classification of the seminiferous epithelium, is conventionally evaluated and staged based on established histological and histopathological criteria, which remain the gold standard for defining developmental cohorts during male gametogenesis (33). As the nucleus gradually condenses during this process, transcriptional activity becomes increasingly restricted. To ensure that proteins required for later stages can still be produced in a timely manner, the corresponding regulatory genes, collectively referred to as spermatogenic genes, must therefore be transcribed in advance and their transcripts are stored as translationally repressed messenger ribonucleoprotein complexes (mRNPs) until translation is activated at the appropriate stage (34).

This developmental context is highly compatible with the concept of biomolecular condensation, because RNA-rich membraneless granules can provide spatially organized compartments for transcript storage, translational repression, and regulated release (35). In mammalian male germ cells, these structures include several morphologically and molecularly distinct RNA granules, such as intermitochondrial cement (IMC), Piwi-interacting RNA (piRNA)-processing granules, and the chromatoid body (CB), each of which is associated with different stages of spermatogenesis and distinct layers of RNA regulation (36–39). Recent reviews and conceptual discussions emphasize that these compartments participate in post-transcriptional RNA regulation, small RNA processing, mRNA storage and protection, translational control, and germ cell fate regulation (36). However, their composition, material properties, and functional requirements remain incompletely resolved. Therefore, although many male germ cell RNA granules are now discussed within the framework of biomolecular condensation, their designation as functional LLPS-driven organelles requires direct evidence linking condensate formation or material properties to a defined biological function.

Primary studies in Drosophila provide examples of how germ granules can regulate post-transcriptional control. Hakes and Gavis showed that germ granules undergo fusion-driven growth and functional remodeling, selectively protecting some mRNAs while promoting the degradation of others, thereby supporting pole cell migration and gonad formation (40). More recently, Ramat et al. demonstrated that the biphasic organization of *Drosophila* germ granules spatially separates translational activation and repression, linking granule architecture to mRNA storage and translation control (41). These findings provide important functional evidence from an invertebrate model, but they should not be directly generalized to mammalian male germ cell RNA granules without additional experimental validation. However, current conceptual discussions caution that not every RNA granule should automatically be interpreted as a functional phase-separated compartment; some condensates may arise as incidental by-products when RNP complexes exceed their solubility threshold. Therefore, functional assignment requires evidence that condensate assembly, material state, or client recruitment is necessary for a defined biological process.

### 2.4 Mechanistic evidence for LLPS in germline RNA regulation and spermatogenesis

In the *C. elegans* germline, Aoki et al. showed that P granule-mediated mRNA repression requires both condensate assembly and localized regulators, including the scaffold protein PGL-1 and the Argonaute protein WAGO-1 (42). Consistently, Wan et al. demonstrated that ZNFX-1- and WAGO-4-containing liquid-like Z granules are spatially organized with P granules and Mutator foci into ordered PZM granule assemblies, thereby coordinating small RNA-mediated transgenerational epigenetic silencing. Together, these studies provide direct evidence that LLPS contributes to germline RNA regulation and epigenetic inheritance in *C. elegans*; however, whether similar mechanisms operate in mammalian germ cells remains to be determined (43).

Together, these observations suggest that LLPS is not merely a structural phenomenon in germ cells, but an organizing principle that may coordinate RNA storage, translational timing, epigenetic regulation, and cellular remodeling during gametogenesis. In mammalian spermatogenesis, several recent studies further support this concept by identifying LLPS-dependent translational and chromatin-regulatory mechanisms in developing male germ cells. In mouse male germ cells, Gao et al. showed that the Tudor domains of Tudor domain-containing 1 (TDRD1) recognize sDMA-modified proteins, whereas its C-terminal coiled-coil domain mediates oligomerization, thereby driving the formation of TDRD1 condensates. TDRD1 is subsequently recruited to the mitochondrial surface by MILI, where it promotes mitochondrial clustering and triggers IMC assembly through phase separation, thereby supporting piRNA biogenesis, transposon repression, and normal spermatogenesis (**Figure 1A**) (44). In mouse late spermatids, Kang et al. found that FXR1 undergoes LLPS to assemble mRNP condensates, which recruit translation factors such as EIF4G3 and poly(A)-binding protein cytoplasmic 1 (PABPC1) to activate the translation of stored mRNAs, thereby supporting normal spermiogenesis (**Figure 1B**) (45). In human and mouse spermatogenic cells, another study showed that CEP112, a key translational regulator during spermatogenesis, also modulates mRNA translation by forming LLPS-mediated ribonucleoprotein condensates. This process involves the recruitment of the essential translation factors EIF4A1 and EEF1A1, as well as the RNA-binding protein hnRNPA2B1, to assemble a translational complex (**Figure 1C**) (46). Furthermore, in mice, Qin et al. showed that, independently of TSLN, coiled-coil glutamate-rich protein 1 (CCER1) forms phase-separated nuclear condensates that coordinate the histone-to-protamine transition during postmeiotic spermatid differentiation, thereby promoting sperm chromatin compaction and male fertility (**Figure 1D**) (47). Notably, human genetic evidence further suggests the potential relevance of CCER1 to male infertility, although the direct mechanistic evidence for LLPS-dependent CCER1 function has mainly been obtained from mouse models.

**Figure 1:**
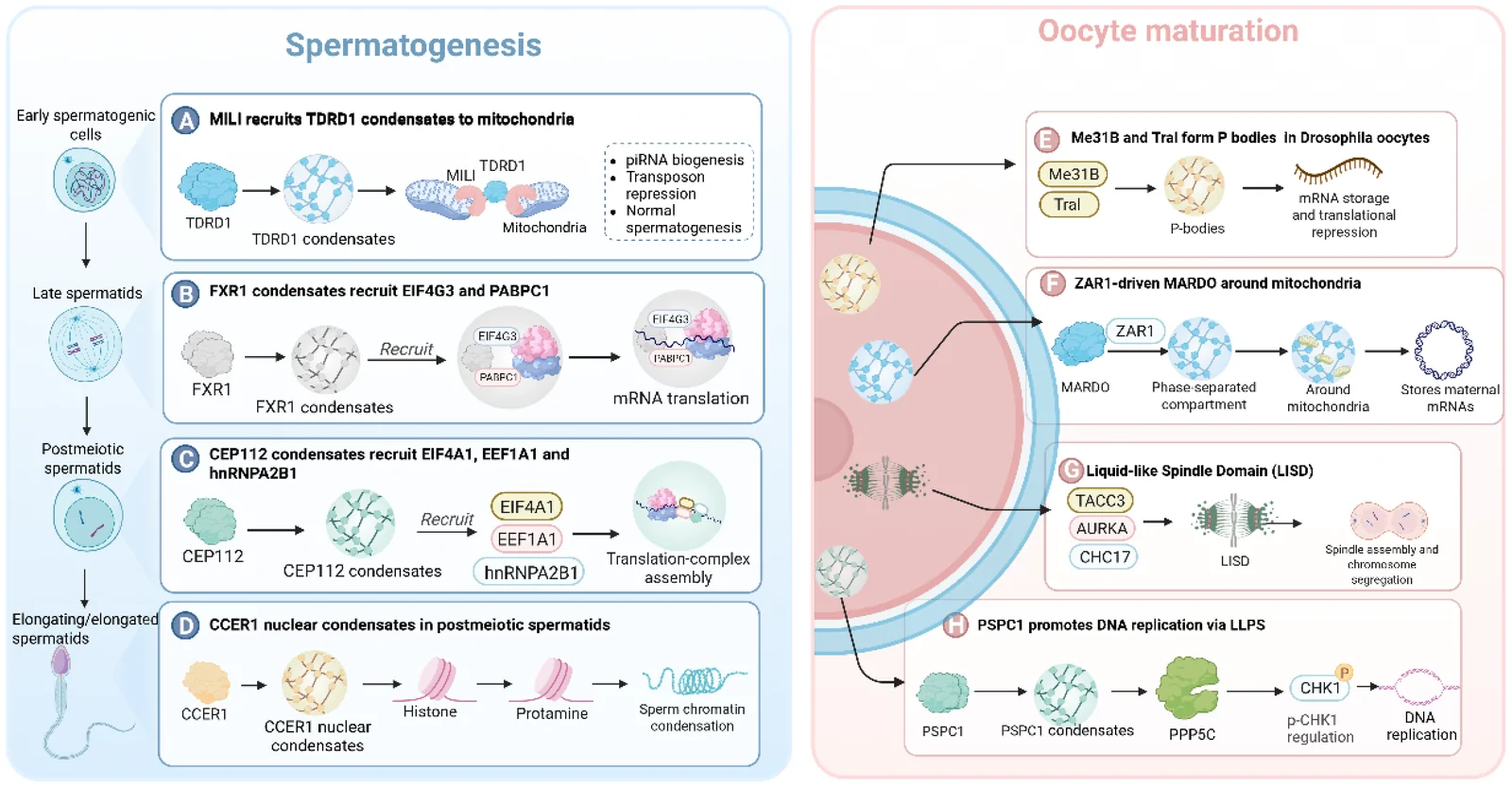
LLPS-mediated biomolecular condensates regulate spermatogenesis and oocyte maturation. LLPS-mediated condensates participate in multiple stages of gametogenesis. **(A–D)** LLPS-associated condensates during spermatogenesis. **(A)** MILI recruits TDRD1 condensates to the mitochondrial surface, facilitating piRNA biogenesis, transposon repression, and normal spermatogenesis. **(B)** FXR1 condensates recruit translation-associated factors, including EIF4G3 and PABPC1, to regulate mRNA translation during spermiogenesis. **(C)** CEP112 condensates recruit EIF4A1, EEF1A1, and hnRNPA2B1 to facilitate the assembly of translation-related complexes during spermatogenesis. **(D)** CCER1 nuclear condensates coordinate the histone-to-protamine transition, promoting sperm chromatin condensation. **(E)** Me31B/Tral-containing P bodies regulate maternal mRNA storage and translational repression in Drosophila oocytes. **(F)** ZAR1-driven MARDOs form phase-separated compartments around mitochondria to store maternal mRNAs, contributing to translational control during oocyte maturation. **(G)** The LISD concentrates TACC3, AURKA, and CHC17, facilitating spindle assembly and chromosome segregation. **(H)** PSPC1 condensates interact with PPP5C to regulate CHK1 phosphorylation and DNA replication during oocyte maturation.

As comparative evidence from a non-mammalian system, Leyden et al. reported that protamines in the spider *Steatoda* sp. are associated with LLPS-related chromatin transitions during spermiogenesis, suggesting that phase-separation-related chromatin remodeling may occur across evolutionarily distant species. However, because this evidence is derived from an arthropod model, it should be interpreted as supportive cross-species evidence rather than direct evidence for mammalian spermatogenesis (48). Together, these primary studies support the involvement of LLPS in selected, experimentally validated layers of spermatogenic regulation, including IMC assembly and piRNA biogenesis, translational control of stored mRNAs, and late-stage chromatin remodeling required for sperm maturation. Importantly, these examples should not be generalized to all male germ cell granules. Consistent with recent conceptual discussions of RNA granules, a clear distinction should be maintained between classical RNA-rich germ granules, condensate-like assemblies, and LLPS-dependent functional compartments whose biological roles have been tested by perturbing condensate formation, material properties, or client recruitment. These experimentally supported examples of LLPS-mediated regulation in spermatogenesis, together with related mechanisms in oocyte maturation, are summarized in **Figure 1**.

### 2.5 LLPS in oocyte maturation and maternal RNA regulation

Oocyte maturation presents a distinctive regulatory challenge: transcriptional activity is largely silenced, while large pools of maternal RNAs and proteins must be selectively stored, stabilized, and translationally regulated to support oocyte maturation and early embryonic development (49). This requirement is broadly conserved across species and creates a strong dependence on cytoplasmic compartmentalization. Emerging evidence suggests that LLPS contributes to this organization, although the molecular components and mechanisms vary considerably among species. Here, non-mammalian models are used to illustrate potentially conserved physical principles, while direct mammalian evidence is emphasized where available.

Maternal mRNA storage and translational repression. Invertebrate studies provide foundational evidence that higher-order RNP organization can protect maternal transcripts and restrain premature translation. In C. elegans, RNP structures aggregate into higher-order particles that help preserve maternal mRNAs during oogenesis (50). In Drosophila, the DEAD-box helicase Me31B and the LSM-family protein Tral contribute to P-body-like condensates that maintain maternal mRNAs in a stored and translationally repressed state (**Figure 1E**) (51). Although these specific molecular components are not strictly conserved in mammals, the underlying principle is directly supported by Cheng et al., who identified the mitochondria-associated ribonucleoprotein domain (MARDO) in mouse oocytes. ZAR1 promotes MARDO assembly around mitochondria, where translationally repressed maternal mRNAs are compartmentalized and stabilized (**Figure 1F**) (52). Thus, invertebrate models provide mechanistic insight into condensate-mediated RNA storage, whereas MARDO provides direct mammalian evidence for a functionally analogous strategy.

Cytoplasmic architecture and organelle organization. The Balbiani body (Bb) is a membraneless structure that concentrates RNAs, proteins, and organelles during vertebrate oogenesis (53). In zebrafish, Woodruff et al. showed that Bb assembly involves LLPS followed by a transition toward a more solid-like state (54), while Kar et al. demonstrated that the intrinsically disordered protein Bucky ball (Buc) promotes condensate maturation under spatial and cytoskeletal constraints (55). These findings illustrate a potentially conserved physical principle whereby condensates can transition from dynamic to stabilized states during oocyte development. However, the specific Buc-dependent mechanism has not been established in mammalian oocytes. Thus, zebrafish provides comparative vertebrate evidence for condensate maturation rather than direct evidence of an equivalent mammalian mechanism.

Spindle assembly and cell-cycle regulation. More direct evidence is available in mouse oocytes. So et al. identified a liquid-like spindle domain (LISD) that concentrates TACC3, AURKA, and CHC17 to promote acentrosomal spindle organization and chromosome segregation (**Figure 1G**) (56). Li et al. further showed that PSPC1 undergoes phase separation and interacts with PPP5C to regulate CHK1 phosphorylation and oocyte cell-cycle progression (**Figure 1H**) (57). Together, these studies indicate that LLPS may contribute not only to maternal RNA storage but also to dynamic structural and signaling processes during mammalian oocyte maturation. Whether analogous LISD- or PSPC1-dependent mechanisms operate in human oocytes remains to be determined.

Collectively, current evidence supports a hierarchical model in which non-mammalian studies define general principles of condensate-mediated RNA regulation and material-state transitions, whereas mouse studies provide direct evidence for LLPS-dependent maternal mRNA compartmentalization, spindle organization, and cell-cycle regulation (52, 56, 57). The strongest conservation therefore appears to be at the level of physical and functional principles rather than identical molecular components. Direct mechanistic evidence in human oocytes remains limited, and the extent to which these condensate mechanisms are conserved in humans requires further investigation.

### 2.6 Putative roles of LLPS in gonadal somatic endocrine cells

Beyond germ cells, the role of LLPS in gonadal somatic cells remains poorly understood. While direct evidence in testicular Leydig cells or ovarian granulosa and luteal cells is still limited, available findings provide indirect evidence suggesting that condensate-associated mechanisms may influence gonadal endocrine function. A key example is the long non-coding RNA NEAT1, which is required for the assembly of paraspeckles—nuclear membraneless bodies formed through phase separation (58). Since Neat1 deficiency has been reported to impair corpus luteum formation and affect pregnancy in mice, NEAT1-dependent paraspeckle organization may be relevant to ovarian endocrine activity, particularly luteal function (59, 60). However, whether this reproductive phenotype is directly caused by altered LLPS-mediated condensate formation remains to be determined, and further studies are needed to establish whether similar mechanisms operate in other steroid-producing gonadal somatic cells. Thus, unlike the increasingly direct evidence available for selected LLPS-dependent processes in mammalian germ cells, the proposed role of phase separation in gonadal endocrine somatic cells remains largely inferential.

### 2.7 Summary of LLPS functions in gametogenesis

Overall, current evidence supports the involvement of LLPS in selected aspects of spermatogenesis and oocyte maturation, particularly RNA metabolism, translational regulation, condensate maturation, chromatin remodeling, spindle organization, and cell cycle control. However, these conclusions should be interpreted according to both species and evidence level. In spermatogenesis, several LLPS-dependent mechanisms have been demonstrated directly in mouse and, in selected cases, human spermatogenic cells. In oocyte biology, by contrast, mechanistic principles of RNA storage and condensate maturation have been derived in part from C. elegans, Drosophila, zebrafish, and other non-mammalian models, whereas direct mammalian evidence is strongest for selected processes demonstrated in mouse oocytes, including maternal mRNA compartmentalization, acentrosomal spindle assembly, and cell-cycle regulation. Therefore, extrapolation from non-mammalian models to mammalian or human reproduction should remain cautious. In particular, direct mechanistic validation of LLPS-dependent processes in human oocytes remains an important unresolved area. In contrast, direct evidence for LLPS-mediated regulation in gonadal somatic endocrine cells remains limited, and its roles in Leydig, granulosa, and luteal cells require further validation.

## 3 LLPS is inextricably linked to gonadal endocrine function and related tumors

Beyond traditional genetic models, LLPS has emerged as an important regulatory framework for understanding certain aspects of tumorigenesis and cancer progression (61). In many cancers, genetic and epigenetic alterations, as well as aberrant expression or mutation of condensate-associated proteins, can perturb the assembly of biomolecular condensates, leading to dysregulation in chromatin remodeling, transcription, and DNA repair (62–64). This may be particularly relevant to sex hormone-dependent reproductive malignancies, where LLPS may serve as a regulatory interface coordinating the interplay between steroid hormone receptors and the genome. Tong et al. also further proposed that LLPS offers new insights into cancer development and may provide a conceptual basis for condensate-targeted therapeutic strategies (65). The following discussion evaluates how these LLPS-mediated mechanisms may contribute to the progression of specific hormone-related reproductive cancers.

### 3.1 The role of LLPS in gonadal endocrine tumors such as ovarian cancer

In estrogen-responsive gynecological malignancies, including subsets of ovarian cancer (OC) and endometrial cancer (EC), E2/ER signaling is a central driver of oncogenesis (66–68). Beyond traditional transcriptional regulation, recent evidence reveals that ligand-activated ERs can participate in LLPS-associated transcriptional condensates with coactivators such as MED1 (27, 69–71). These phase-separated transcriptional complexes may facilitate the assembly of molecular hubs that amplify hormone-responsive gene expression. However, the extent to which ER-associated condensates directly drive OC or EC progression remains incompletely defined and requires tumor-context-specific validation.

Qiu et al. discovered that differential expression of LLPS-associated genes in OC is involved in multiple cancer-related pathways, including the MAPK signaling pathway, cell cycle regulation, and DNA replication. Through comprehensive analysis of multiple datasets, they constructed and validated an 11-gene LLPS-related prognostic model, including EIF3J, BYSL, NRGN, SAP18, PACSIN2, DUSP10, EIF6, HMBOX1, UTP3, HOMER2, and KIAA0355, as a prognostic model for OC (61). Although such signatures may capture clinically relevant heterogeneity, their component genes are not mechanistically equivalent. Conceptually, LLPS-associated genes can be divided into direct condensate drivers, indirect modulators, and disease-associated markers. Assignment to the first category requires evidence that a molecule promotes condensate assembly or alters condensate composition, dynamics, or material properties in cells. For example, EIF6 and HMBOX1 have been associated with OC progression (72, 73). But current evidence does not demonstrate that they directly nucleate condensates or function as multivalent organizers of LLPS in OC cells. Therefore, these genes are more appropriately interpreted as LLPS-associated biomarkers or candidate regulators rather than validated condensate drivers. This distinction is important because prognostic association and differential expression alone cannot establish an LLPS-driven mechanism; direct biophysical or live-cell evidence is required to link condensate integrity to tumor phenotypes.

By contrast, some recent studies in OC provide stronger mechanistic support for a causal role of LLPS. Zhan et al. reported that high lysine acetyltransferase 6A (KAT6A) levels promote PARP inhibitor resistance in OC independently of its catalytic activity. Mechanistically, apurinic/apyrimidinic endonuclease 1 (APEX1) facilitates KAT6A LLPS, and KAT6A condensates interact with PARP1 to impair PARP inhibitor-induced PARP1 trapping, thereby reducing PARPi cytotoxicity (74). This study establishes a clearer mechanistic chain from condensate remodeling to altered DNA repair-associated drug response, suggesting that modulation of KAT6A-dependent condensates may help restore PARP inhibitor sensitivity. Consistent with this concept, Mobet et al. recently showed that A-kinase anchoring protein 8 (AKAP8) is highly expressed in OC and predicts poor prognosis. AKAP8 is enriched at chromatin and undergoes phase separation to regulate the transcription of the short isoform of heterogeneous nuclear ribonucleoprotein U-like 1 (hnRNPUL1), thereby promoting OC cell proliferation, metastasis, and disease progression (75). Liu et al. further discovered that 5-methylcytosine (m5C) modification of E2F1 mRNA promotes LLPS of Y-box binding protein 1 (YBX1), thereby enhancing E2F1 expression and driving tumorigenesis (76). In addition to transcriptional control, LLPS has also been implicated in translational regulation in OC. Xiong et al. showed that m6A-modified RNAs and insulin-like growth factor 2 mRNA-binding protein 2 (IGF2BP2) acetylation facilitate IGF2BP2 LLPS, leading to enhanced *de novo* protein synthesis and OC progression (77). Beyond translational regulation, Zuo et al. further demonstrated that serine/arginine-rich splicing factor 9 (SRSF9) forms phase-separated condensates within the nucleus, where it recognizes non-m6A-modified NUMB mRNA and promotes an oncogenic alternative splicing switch (78). A non-coding RNA transcribed from the promoter region of phosphatidylinositol-4,5-bisphosphate 3-kinase catalytic subunit beta (PIK3CB) is generated by RNA polymerase II (RNA Pol II). This ncRNA further recruits heterogeneous nuclear ribonucleoprotein L (hnRNPL) and promotes its LLPS, thereby activating PIK3CB transcription and ultimately driving cancer progression (79). Beyond transcriptional and RNA-processing programs, LLPS has also been implicated in intercellular communication and metastatic dissemination in OC. Wu et al. showed that chromobox 2 (CBX2) forms nuclear condensates through phase separation, upregulates FLOT1 expression, and promotes lipid raft remodeling, thereby enhancing lipid raft-mediated extracellular vesicle (EV) secretion and ultimately promoting ovarian cancer cell migration, invasion, and omental metastasis (80). Together, these studies indicate that LLPS contributes to OC progression through multiple mechanistic axes, including PARP inhibitor resistance, transcriptional regulation, RNA modification-dependent condensate assembly, translational control, alternative splicing, metabolic reprogramming, and EV-mediated metastatic communication. Nevertheless, these pathways should not be treated as a uniform mechanistic class: some represent experimentally supported condensate-driven mechanisms, whereas others remain candidate regulatory axes requiring further functional validation.

Wang et al. employed multi-omics analysis to evaluate the expression patterns, heterogeneity, prognostic value, and immunological significance of LLPS-related genes in EC, and established a prognostic model comprising EIF2S2, SNRPC, PRELID1, and NDUFB9 (81). Although these genes have been implicated in tumor-related processes, including MYC-associated oncogenic signaling, epithelial-mesenchymal transition, mitochondrial reactive oxygen species regulation, and mitochondrial metabolism (82–85), their inclusion in an LLPS-associated prognostic model should not be taken as evidence that each gene directly governs condensate formation in EC. At present, these genes are better interpreted as LLPS-associated indicators of tumor state rather than validated condensate drivers, because direct evidence that they nucleate condensates or control condensate dynamics in EC cells is lacking. This distinction is important for avoiding overinterpretation of prognostic association as functional proof. In contrast, Lv et al. recently provided more direct mechanistic evidence that 2,5-MeC induces EIF1AX nucleolar translocation in EC cells. Once localized to the nucleolus, EIF1AX undergoes LLPS through its N- and C-terminal intrinsically disordered regions and recruits DDX21 to form EIF1AX-DDX21 nucleolar condensates. These condensates suppress rDNA transcription and pre-rRNA production, thereby inducing cellular senescence and increasing the sensitivity of EC cells to senolytic therapy (86).

### 3.2 The role of LLPS in estrogen-related tumors such as breast cancer

Previous studies have indicated that progesterone signaling may contribute to the development and recurrence of BC, particularly in hormone-responsive contexts, while E2 may further increase the risk of BC by enhancing progesterone signaling (87). As discussed above, ER-mediated LLPS also contributes to E2-induced gene expression. From a clinical perspective, endocrine therapy remains a cornerstone of treatment for hormone receptor-positive BC, although treatment-associated adverse effects represent an important challenge in long-term disease management. For example, Zhu et al. systematically evaluated acupuncture for aromatase inhibitor-related arthralgia in patients with BC, highlighting the clinical importance of managing endocrine therapy-associated complications alongside sustained anticancer treatment (88). This clinical dimension complements emerging mechanistic efforts to understand how hormone-responsive tumor cells maintain oncogenic signaling and acquire therapeutic vulnerabilities.

Beyond steroid hormone receptor-centered condensates, RNA molecules, particularly long non-coding RNAs (lncRNAs), have also emerged as important regulators of LLPS-dependent oncogenic programs in BC. In recent years, researchers have increasingly focused on the role of lncRNAs in tumor progression. Li et al. showed that small nucleolar RNA host gene 9 (SNHG9) interacts with large tumor suppressor kinase 1 (LATS1) and promotes its phase separation, thereby suppressing LATS1-mediated phosphorylation of YAP and facilitating tumorigenesis (89). Wang et al. reported that RUNX1 intronic transcript 1 (RUNX1-IT1) interacts with insulin-like growth factor 2 mRNA-binding protein 1 (IGF2BP1) and promotes the formation of IGF2BP1 condensates, thereby enhancing glutathione peroxidase 4 (GPX4) expression and promoting BC progression (90). Given the central role of GPX4 in restraining lipid peroxidation and ferroptotic cell death, the LLPS-dependent regulation of GPX4 provides a mechanistic link between biomolecular condensate biology and the broader regulatory network governing ferroptosis. In a mechanistically relevant but non-malignant disease context, Yang et al. recently demonstrated that tamarixetin suppresses neuronal ferroptosis through activation of nuclear factor erythroid 2-related factor 2 (Nrf2)-dependent antioxidant signaling (91). Although these findings were obtained in models of ischemic stroke rather than cancer, they provide complementary evidence for the therapeutic tractability of ferroptosis-regulatory pathways. Whether LLPS directly intersects with Nrf2-dependent ferroptosis regulation in hormone-responsive BC remains to be established. These studies suggest that certain lncRNAs may function not merely as passive transcripts, but as organizers or stabilizers of oncogenic condensates. At the same time, it should be recognized that not every lncRNA associated with LLPS necessarily serves as a condensate driver; some may instead reflect broader hormone-responsive transcriptional states. Thus, direct evidence linking a given lncRNA to condensate assembly, composition, or function remains essential when assigning mechanistic significance. In addition to endogenous RNA-mediated condensates, engineered modulation of RNA phase behavior may also have therapeutic relevance. Xing et al. reported that cationic polymers (CPs) can induce mRNA LLPS in tumor cells and suppress BC progression (92), refining the therapeutic potential of LLPS by demonstrating that modulation is not restricted to blocking endogenous condensates; rather, engineered shifts in RNA phase behavior can actively drive antitumor responses. This shifts the focus toward a more nuanced pharmacological approach, where the objective—whether to dismantle, reprogram, or redirect condensate function—dictates the specific intervention strategy.

Several studies have attempted to construct multigene risk-scoring models based on LLPS-associated genes to predict BC prognosis (61). However, the prognostic value of LLPS-related signatures remains controversial. Qing et al. found that their predictive performance in BC was limited (93). Xie et al. later established a multi-omics prognostic model incorporating POLR3GL, PLAT, NDRG1, HMGB3, HSPH1, PSMD7, PDCD2, NONO, and PGAM1 (94). Wang et al. further developed a machine learning-based LLPS prognostic model (MDLS) to improve personalized prognostic assessment in BC (95). While LLPS-associated signatures may capture biologically relevant heterogeneity, their clinical utility must be balanced against a lack of mechanistic evidence. Prognostic correlation is not synonymous with functional dependence; thus, a clear distinction must be maintained between predictive biomarkers and validated molecular regulators. Within the current BC landscape, these models function primarily as stratification tools rather than definitive evidence of altered condensate biology.

Additionally, Lu and colleagues found that phosphorylated histone deacetylase 6 (phospho-HDAC6) undergoes LLPS with 14-3-3θ, thereby driving aberrant chromatin architecture, distinct histone acetylation patterns, and transcriptional reprogramming, ultimately promoting tumorigenesis (96, 97). This study links a comprehensive molecular axis by linking a specific post-translational modification directly to condensate assembly, epigenetic remodeling, and oncogenic transcription. Beyond mere correlation, it points to several potential intervention levels, suggesting that intervention can be precisely targeted at the upstream regulatory pathway, the phospho-dependent interaction interface, or the structural integrity of the pathogenic condensate. More recently, Xie et al. found that forkhead box protein M1 (FOXM1) undergoes LLPS with FKH consensus DNA elements and sustains oncogenic transcriptional programs required for metastatic outgrowth. AMPK agonists inhibit FOXM1 condensation and reduce oncogenic transcription, suggesting that FOXM1 condensates may represent a potential therapeutic vulnerability in breast cancer (98). In addition to directly targeting pathogenic condensates, LLPS may also influence the intracellular behavior of anticancer agents. Klein et al. demonstrated that small-molecule cancer therapeutics can selectively partition into distinct nuclear condensates, and that such condensate partitioning can affect drug activity (99). Together, these findings extend the therapeutic relevance of LLPS from condensate-targeted intervention to condensate-aware drug design.

### 3.3 The role of LLPS in androgen-related tumors such as prostate cancer

While the AR pathway remains the central driver of prostate cancer (PCa), its reactivation—through both ligand-dependent and ligand-independent mechanisms—underpins the transition to castration-resistant prostate cancer (CRPC), an advanced and currently incurable disease state (100–102). Emerging evidence suggests that this persistent signaling is increasingly governed by LLPS, a mechanism that organizes transcription factors into dynamic hubs to amplify gene expression (2, 69). Given that several nuclear hormone receptors utilize phase separation for transcriptional control, identifying how aberrant AR condensates contribute to drug resistance offers a promising avenue for uncovering new therapeutic vulnerabilities in CRPC (103, 104).

The AR protein comprises an N-terminal domain (NTD), a DNA-binding domain (DBD), a hinge region, and a ligand-binding domain (LBD) (105, 106). When PCa progresses to advanced CRPC, the efficacy of LBD-targeting agents declines, partly because splice variants such as AR-V7 lack the LBD (107). Research indicates that AR, including AR-V7, can interact with RNA through the DBD and mediate RNA-dependent LLPS (108). In addition, the NTD itself exhibits phase-separation capacity (109), and full-length AR also undergoes LLPS that can be inhibited by antagonists targeting different AR domains. Certain antiandrogens may even promote LLPS of mutant AR variants in the absence of androgen, whereas a small-molecule compound that suppresses AR LLPS inhibits the growth of drug-resistant PCa cells *in vivo* (30). These observations support a more mechanistic interpretation of AR-driven condensates: multiple AR domains appear to contribute cooperatively to condensate assembly, and persistence of condensate-forming capacity despite loss of the LBD may help explain why transcriptional output can be maintained in CRPC. This also makes LLPS-directed intervention conceptually distinct from conventional receptor antagonism, because the therapeutic goal is not only to block ligand binding, but also to disrupt the higher-order assemblies that sustain aberrant transcription. In addition to AR itself, RBM14 promotes phase separation under androgen-deprived conditions, forming condensate-like assemblies that sustain AR-dependent transcriptional output and maintain PSA expression in human prostate cancer cells. This mechanism suggests that RBM14-associated condensates may represent a potential vulnerability in advanced or therapy-resistant PCa (110).

In PCa, oncogenes are frequently activated through super-enhancers (SEs), where transcription factors and coactivators containing disordered regions form LLPS-related condensates (111). AR forms condensates with MED1 at SEs, thereby promoting oncogene expression (30). OCT4 can also participate in LLPS-mediated SE regulation: in AR-positive PCa, OCT4 cooperates with AR and FOXA1, whereas in AR-negative PCa, OCT4 forms condensates with NRF1 to drive alternative oncogenic transcriptional programs (107). Similarly, LSD1 forms an LLPS-related network with FOXA1 and BRD4 at SEs (112). These studies identify LLPS at SEs as a spatial amplifier of lineage-specific transcription. However, the potential for global condensate disruption to inadvertently impair tumor-suppressive circuits highlights a major hurdle in therapeutic selectivity (113). Consequently, distinguishing between candidate core condensate scaffolds such as AR and context-dependent organizers or clients is essential for establishing mechanistic and therapeutic priority.

LLPS also coordinates non-transcriptional adaptations in PCa, particularly during stress-induced progression. For instance, DEAD-box helicase 3 X-linked (DDX3) undergoes phase separation with AR mRNA and stress granule-associated PABP1 to repress AR translation, facilitating transition toward a castration-resistant phenotype (114). This mRNA-centric regulation is further nuanced by m6A modifications: m6A-tagged AR mRNA undergoes LLPS with the reader YTHDF3, whereas unmodified transcripts associate with G3BP1 under stress. By differentially partitioning AR mRNA into distinct condensates, these events fine-tune AR protein levels to ensure tumor cell survival under adverse conditions (115). These findings broaden the known scope of LLPS in PCa, indicating that its role extends beyond transcriptional activation to include post-transcriptional stress adaptations. Crucially, the spatial partitioning of AR mRNA into different condensates dictates its translational fate, establishing a direct link between RNA modifications and adaptive protein synthesis in therapy resistance.

Speckle-type POZ protein (SPOP), a substrate adaptor of the Cullin3-RING ubiquitin ligase complex, is frequently mutated in human cancers, particularly PCa (116). Under physiological conditions, SPOP phase-separates with DAXX to facilitate its ubiquitination and subsequent degradation, thereby inducing apoptosis. However, cancer-associated SPOP mutations impair this assembly, stabilizing DAXX and ultimately driving tumorigenesis (109). SPOP provides a compelling counter-example to the “all-oncogenic” view of LLPS, demonstrating that phase-separated assemblies can be essential for tumor suppression. This functional duality cautions against indiscriminate LLPS inhibition, as broad disruption might ablate protective physiological condensates. Accordingly, therapeutic strategies must distinguish between the pathogenic gain and physiological loss of condensate function, shifting the focus toward context-specific intervention. The clinical potential of targeting phase behavior is further demonstrated by AR-V7, a variant that lacks LBD but retains its capacity for LLPS-mediated transcription. By exploiting this vulnerability, Thiyagarajan et al. utilized the irreversible antagonist SARICA to disrupt AR transactivation domain-driven condensates, achieving marked efficacy in advanced PCa models (117). This strategy reveals that even LBD-deficient tumors remain dependent on condensate-mediated organization, shifting the therapeutic focus from traditional hormone antagonism to the direct perturbation of oncogenic phase behavior. To provide an integrated overview of these findings, **Table 2** summarizes the LLPS-associated biomolecules, related condensates, biological functions, and corresponding cancer types discussed above.

**Table 2:** Summary of LLPS-associated biomolecules, related condensates, and biological functions in hormone-related reproductive tumors.

Cancer type	Biomolecule involved in LLPS	Related condensates	Biological role of biomolecules	Reference
Ovarian cancer	EIF3J, BYSL, NRGN, SAP18, PACSIN2, DUSP10, EIF6, HMBOX1, UTP3, HOMER2, KIAA0355	Not defined	Prognostic stratification through LLPS-related gene programs	Qiu Y et al., 2021 (61)
	KAT6A, APEX1, PARP1	KAT6A condensates	Promotion of PARP inhibitor resistance.	Zhan Z et al., 2024 (74)
	AKAP8, hnRNPUL1	AKAP8 chromatin-associated condensates	Activation of pro-proliferative and pro-metastatic transcriptional programs	Mobet Y et al., 2024 (75)
	m5C-modified E2F1 mRNA, YBX1	YBX1 condensates	Enhancement of E2F1-driven tumorigenesis	Liu X et al., 2024 (76)
	m6A-modified RNAs, acetylated IGF2BP2	IGF2BP2 condensates	Promotion of protein synthesis and tumor progression	Xiong X et al., 2025 (77)
	SRSF9, non-m6A-modified NUMB mRNA	SRSF9 nuclear condensates	Induction of oncogenic alternative splicing programs	Zuo X et al., 2025 (78)
	PIK3CB promoter-derived ncRNA, hnRNPL	hnRNPL condensates	Activation of PIK3CB transcription, glycolysis, and cancer progression	Qin F et al., 2025 (79)
	CBX2, FLOT1	CBX2 condensates	Promotion of lipid raft remodeling, extracellular vesicle secretion, and metastasis	Wu Q et al., 2026 (80)
Endometrial cancer	EIF2S2, SNRPC, PRELID1, NDUFB9	Not defined	Prognostic stratification through LLPS-related gene programs	Wang J et al., 2022 (81)
	EIF1AX, DDX21	Nucleolar condensates	Suppression of rRNA biogenesis and induction of cellular senescence	Lv C et al., 2026 (86)
Breast cancer	SNHG9, LATS1, YAP	LATS1 condensates	Activation of YAP-dependent tumorigenic signaling	Li RH et al., 2021 (89)
	RUNX1-IT1, IGF2BP1, GPX4	IGF2BP1 condensates	Promotion of breast cancer progression through GPX4-mediated ferroptosis suppression	Wang S et al., 2023 (90)
	mRNA, cationic polymers	mRNA condensates	Therapeutic suppression of tumor growth through engineered RNA condensation	Xing Z et al., 2022 (92)
	POLR3GL, PLAT, NDRG1, HMGB3, HSPH1, PSMD7, PDCD2, NONO, PGAM1	Not defined	Prognostic stratification through LLPS-related biomarker panels	Xie J et al., 2023 (94)
	Phosphorylated HDAC6, 14-3-3θ	Phospho-HDAC6 condensates	Induction of chromatin remodeling and oncogenic transcriptional reprogramming	Lu B et al., 2024 (97)
	FOXM1, FKH consensus DNA elements	FOXM1–DNA condensates	Maintenance of metastatic transcriptional programs	Xie F et al., 2025 (98)
	MED1, ERα	MED1/ERα transcription condensates; SEs	Regulation of tamoxifen response and endocrine therapy resistance through condensate-dependent drug partitioning	Klein IA et al., 2020 (99)
Prostate cancer	AR, RNA, AR NTD and DBD	Transcription condensates	Maintenance of AR-driven transcriptional output in castration-resistant prostate cancer	Zhang F et al., 2023 (30); Ahmed J et al., 2021 (108); Bouchard JJ et al., 2018 (109)
	RBM14 and AR pathway-associated factors	RBM14 condensates	Sustaining AR signaling under androgen-deprived conditions	Tsuji K et al., 2023 (110)
	AR, MED1	SEs	Amplification of super-enhancer-associated oncogenic transcription	Zhang F et al., 2023 (30)
Prostate cancer	OCT4, AR, FOXA1, NRF1	Transcription factor/enhancer condensates	Activation of AR-dependent and AR-independent oncogenic transcriptional programs	Takayama KI et al., 2021 (107)
	LSD1, FOXA1, BRD4	SEs	Activation of super-enhancer-driven oncogenic transcription	Li M et al., 2023 (112)
	DDX3, AR mRNA, stress-granule-associated factors	SG-related RNP condensates	Post-transcriptional repression of AR protein expression and promotion of castration-resistant transition	Vellky JE et al., 2020 (114)
	m6A-modified AR mRNA, YTHDF3, G3BP1	SGs	Stress-adaptive Regulation of AR mRNA translation and tumor cell survival under therapeutic stress	Somasekharan SP et al., 2022 (115)
	SPOP, DAXX	Nuclear speckles/SPOP-associated nuclear condensates	Degradation of oncoproteins and suppression of tumorigenesis	Bouchard JJ et al., 2018 (109)
	AR-V7, SARICA	AR-V7 transcription condensates	Maintenance of AR-V7 transcriptional activity and therapeutic vulnerability	Thiyagarajan T et al., 2023 (117)

While LLPS research has gained momentum in OC, EC, BC, and PCa, its role in other gonadal and endocrine malignancies remains largely unexplored—a gap likely driven by a combination of low disease incidence and a lack of specialized experimental models. To advance the field, future efforts must transcend descriptive cataloging and establish a rigorous functional hierarchy that distinguishes bona fide condensate drivers from indirect regulators and associated biomarkers. Prioritizing direct biophysical and live-cell validation over simple transcriptomic correlation is essential to isolate therapeutically actionable vulnerabilities from broad disease signatures. Such a transition from conceptual observation to mechanistic rigor will be pivotal for translating LLPS biology into precise clinical interventions for reproductive tumors.

## 4 Summary and future outlook

LLPS has reshaped our understanding of cellular organization, transitioning from a biophysical phenomenon to a fundamental regulatory layer in reproductive biology. Beyond maintaining germ cell homeostasis, dysregulated LLPS may provide a versatile scaffold that orchestrates the transcriptional and post-transcriptional dysregulation driving tumor initiation and therapeutic resistance.

In the male reproductive system, LLPS is known to regulate specific aspects of spermatogenesis, RNA metabolism, and chromatin remodeling. Its oncogenic role is most defined in PCa, where AR-containing condensates sustain signaling in castration-resistant states. Conversely, in female reproductive tissues, LLPS intersects with estrogen-mediated signaling to drive gynecologic malignancies. Despite these advances, mechanistic evidence across diverse sex hormone-dependent tumors remains uneven. The field now faces a critical transition: moving beyond the descriptive cataloging of LLPS-associated factors toward a functional hierarchy that distinguishes bona fide condensate drivers from secondary markers or adaptive responses.

Future research should prioritize three pillars: (i) defining the precise molecular determinants of condensate assembly; (ii) establishing rigorous validation criteria to separate pathogenic condensates from physiological ones; and (iii) identifying druggable vulnerabilities within LLPS-mediated networks. Achieving a high-resolution understanding of these phase-separated interfaces will be pivotal for the mechanistic classification of reproductive tumors and the development of next-generation, rationally targeted therapies.
